# Addressing the day–night divide

**DOI:** 10.1093/jxb/erag239

**Published:** 2026-06-24

**Authors:** Lorna McAusland, Erik H Murchie

**Affiliations:** Division of Plant and Crop Sciences, Sutton Bonington, University of Nottingham, Loughborough LE12 5RD, UK; Division of Plant and Crop Sciences, Sutton Bonington, University of Nottingham, Loughborough LE12 5RD, UK

**Keywords:** Crops, heat, night, photosynthesis, respiration, wheat

## Abstract

This article comments on:

**Shahi PR, Scafaro AP, Thistlethwaite RJ, Atkin OK, Trethowan RM, Rader R, Burns A, Coast O.** 2026. Wheat leaf dark respiration acclimates more strongly at night than in the day when responding to nocturnal warming. Journal of Experimental Botany **77**, 3777–3792. https://doi.org/10.1093/jxb/erag106

This article comments on:

**Shahi PR, Scafaro AP, Thistlethwaite RJ, Atkin OK, Trethowan RM, Rader R, Burns A, Coast O.** 2026. Wheat leaf dark respiration acclimates more strongly at night than in the day when responding to nocturnal warming. Journal of Experimental Botany **77**, 3777–3792. https://doi.org/10.1093/jxb/erag106

**Nocturnal crop physiology to improve climate resilience has been overlooked, in contrast to daytime plant responses. This is becoming a critical omission because nights are warming faster than days in important global agricultural regions, negatively impacting yields. For some of our major crops, such as wheat, these yield reductions necessitate a drive for identifying and understanding key nocturnal processes which not only support whole-crop function throughout the diurnal cycle but also promote sustainable yields under warming nights. One of these vital, temperature-sensitive processes is respiration. In their study, Shahi *et al.* investigate day and night-time rates of respiration in response to nocturnal warming across a historic selection of Australian wheat cultivars. They demonstrate a disparity in the ability of daytime respiration to acclimate to warmer nights and highlight potential avenues for improving wheat resilience to climate change.**

Anthropogenic climate change is driving asymmetric increases in land surface temperatures, with nocturnal warming advancing ∼1.4-fold faster than daytime warming (Davy *et al*., 2017; Cox *et al.*, 2020). This disparity has significant consequences for agricultural productivity in many crop species. Wheat, our most widely cultivated crop, grown across 217 Mha globally, suffers yield losses of 2–10% for every 1 °C rise in minimum (nocturnal) temperature (Lobell and Ortiz-Monasterio, 2007; Sadok and Jagadish, 2020). While the combined effects of extreme daytime (maximum) temperature and rainfall account for up to 40% of annual variation in global grain production (Zampieri *et al*., 2017), recent evidence suggests that night-time temperatures alone may be responsible for 40% of this variation (Schulthess *et al*., 2026, Preprint). This is approximately equivalent to 337 Mt and comparable with the combined annual production of the EU, China, and the USA (USDA, 2025).

Australia exemplifies this emerging threat. Mean temperatures have risen by 1.6 °C between 2011 and 2020—roughly 1.4 times the global average—with eight of the nine warmest years on record occurring since 2013, and high night-time temperature events now occurring five times more frequently than 30 years ago (Grose *et al.*, 2023).

Against this backdrop, Shahi *et al.* (2026) provide a thorough and timely investigation into how Australian wheat cultivars, spanning over a century of breeding history, respond to nocturnal warming at the level of leaf gas exchange. Critically, the study discusses photosynthesis and respiration (here defined as leaf net CO_2_ release) not as independent processes, but as a functionally integrated system with diurnally distinct regulatory strategies within the context of nocturnal warming. Their findings contribute to our understanding of the day–night divide.

## Divergent acclimation of day and night respiration under nocturnal warming

Yield losses under warm nights have conventionally been attributed to an imbalance in two concurrent processes: elevated nocturnal respiration increasing carbon expenditure, and reduced daytime photosynthetic capacity, as a result of diurnal heat damage, constraining carbon supply. It is commonly assumed that temperature increases (day or night) lead to proportionally higher respiration rates, driven by increased metabolic demand in cellular maintenance (and repair) and metabolic turnover (O’Leary *et al*., 2019).

The authors find that temperature-normalized nocturnal respiration (*R*_night_) consistently declined under warm nights, a response indicative of respiratory acclimation, while daytime respiration (*R*_day_) increased. This divergence in response implies different metabolic controls operating during the day or night, with perturbations due to nocturnal warming perhaps resulting in longer term implications for yield. The authors attribute this to the distinct substrate sources available to mitochondrial respiration at night versus during the day: nocturnal respiration draws predominantly on starch remobilized via circadian-controlled degradation, while daytime respiration (including photorespiration) could be more tightly coupled with photosynthesis and therefore less free to down-regulate.

Acclimation of *R*_night_ to nocturnal warming (low- to mid-twenties) is consistent with earlier reports in wheat from McAusland *et al.* (2023) (2 °C above ambient) and Impa *et al*. (2018) (26 °C day/23 °C night). However, conflicting evidence also exists for crops including examples of wheat (26 °C day/23 °C night), rice (29 °C day/26 °C night or ∼3 °C above ambient, Dong *et al.*, 2014; Peraudeau *et al.*, 2015), cotton (32 °C day/30 °C night, Loka and Oosterhuis, 2016), maize (∼3 °C above ambient), soybean (32 °C day/28 °C night, Sankarapillai *et al.*, 2025), sorghum (27°C day/26°C night, Manunta and Kirkham, 1996), and sunflower (27 °C day/26 °C night, Manunta and Kirkham, 1996), where *R*_night_ increased under warm nights. These discrepancies are likely to reflect differences in the type, magnitude, duration, and growth stage of imposed warming—with extreme or sustained high night-time temperatures (consistently >25 °C) perhaps exceeding the capacity for acclimation and advancing severe stress responses. The distinct threshold nature of these responses between crops warrants more systematic investigation across species and warming regimes, with genotypic variation in magnitude of acclimation perhaps a good indicator of yield resilience to warmer nights.

In leaves, ∼75% of leaf nitrogen (N) content is associated with photosynthetic processes (Evans and Clarke, 2019). Similarly, leaf or foliar N has also emerged as a significant predictor of *R*_night_, consistent with the well-established N–respiration scaling relationship and the dependence of mitochondrial capacity on nitrogen-rich enzymes. However, foliar N accounted for only ∼50% of *R*_night_ variation, indicating that other factors including substrate availability, soluble carbohydrate concentrations, and enzyme-level regulation also contribute. The positive scaling of leaf N with *R*_night_ under warming nevertheless strengthens the case for incorporating foliar N as a dynamic input parameter for *R*_night_ in crop and Earth system models—an improvement with broad implications for terrestrial carbon cycle projections.

## Measurement considerations and controlled-environment limitations

An important caveat on the results of this article concerns recent findings on the temporal dynamics of *R*_night_. Practical and logistical constraints (e.g. staff availability, dewfall) mean that most measurements are made at the start of the nocturnal period, yet *R*_night_ has been shown to decline by ∼25% over an 8 h night at constant temperature (Bruhn *et al*., 2022). The magnitude of this decline is also influenced by light environment and *R*_night_ at the beginning of the night period (Jones *et al.*, 2024), again highlighting an inherent day–night link. Leaf-level *R*_night_ also varies with developmental stage, increasing during grain filling as temperatures rise and senescence occurs. Bruhn *et al.* (2022) further demonstrated that temperature accounts for <50% of *R*_night_ variation, underscoring that single time-point measurements and temperature-centric interpretations risk misrepresenting the true magnitude and overlooked dynamic drivers of nocturnal carbon loss. Future studies should integrate continuous monitoring across the nocturnal period to better contextualize these dynamics to identify responses which improve nocturnal resilience.

A further consideration is the distinction between controlled-environment and field conditions. In this study, plants experienced a constant temperature differential between day and night, whereas field nocturnal warming typically manifests as a gradual increase across the diurnal temperature profile. Whether acclimation responses, particularly the degree of *R*_night_ down-regulation, are equivalent under constant versus fluctuating elevated night temperatures remains unclear.

## Photosynthetic stability and shifting biochemical capacity across breeding history

Operational rates of net carbon assimilation at a common temperature (*A*_net_^25^) showed no systematic improvement across a historic cultivar panel (1901–2012), suggesting that historical breeding for yield has not inadvertently enhanced photosynthetic rate. More importantly for climate adaptation, *A*_net_^25^ was broadly maintained under warm nights across nine of the 10 cultivars tested. This stability of *A*_net_^25^ under warming, also observed in McAusland *et al.* (2023), represents an important physiological buffer: even without constructive thermal acclimation of photosynthesis (i.e. an increase in both *A*_net_^25^ and its temperature optimum), the ability of a genotype to avoid photosynthetic decline under warm nights may contribute to yield stability in future climates.

However, the stability of *A*_net_^25^ masks divergent trends in underlying biochemical capacity. *V*_cmax_ and *J*_max_ both declined in more modern cultivars under warm nights, despite no reduction in leaf N. This could imply a shift in N allocation away from photosynthetic proteins such as Rubisco and electron transport components in modern germplasm. The authors suggest that maintained *A*_net_^25^ at reduced protein investment may reflect greater intrinsic efficiency of the photosynthetic machinery in modern cultivars (e.g. reduced maintenance costs for Rubisco turnover and synthesis). The increased *J*_max_/*V*_cmax_ ratio observed under nocturnal warming in modern cultivars further points to a decoupling of ribulose bisphosphate (RuBP) regeneration and carboxylation capacity, suggesting early steps of the Calvin cycle emerging are still critical targets for improving photosynthetic resilience to warming.

## Conclusions and broader context

Nocturnal warming does not influence singular plant metabolic processes in isolation. Warmer nights are climatically coupled to warmer days, meaning that any respiratory or photosynthetic response measured under warm nights will also integrate the metabolic consequences of elevated daytime temperatures (Posch *et al.*, 2022) including photoinhibition, protein damage, and associated repair costs. Anthesis is among the most thermally sensitive stages of wheat development, and even modest increases in night-time temperature can shorten the grain-filling period, alter carbon and N remobilization, and compromise grain quality. Additionally, warmer nights are frequently associated with reduced atmospheric vapour pressure deficits at dusk, transitioning to greater evaporative demand through the day—a combined warmer and drier night environment whose integrated physiological consequences remain less well characterized in crops.

Several priorities emerge from this work that require attention. First is the parameterization and inclusion of representative *R*_night_ in crop and Earth system models. Current frameworks that equate *R*_day_ and *R*_night_—or apply a single temperature response function—could potentially and systematically overestimate night-warming-induced respiratory carbon losses (Bruhn, 2023). Distinct temperature sensitivity functions for *R*_day_ and *R*_night_, incorporating capacity for acclimation, are also needed, along with explicit temperature thresholds and perhaps the proposal of separate thermal optima for day and night respiration rather than a single value. Continuous measurement of *R*_night_ across the nocturnal period combined with light interception data could substantially improve parameterization fidelity. Finally, greater experimental emphasis on biomass and grain-level outcomes, measured alongside leaf physiology, will help resolve which of the identified physiological relationships most effectively predict yield resilience and therefore represent the most productive breeding targets.
